# Pathogenetic characteristics of infectious diarrhea in Yantai City, Shandong Province, 2018–2019

**DOI:** 10.3389/fpubh.2023.1195118

**Published:** 2023-07-06

**Authors:** Qiao Gao, Hong Liu, Weixia Yu, Zhaolin Wang, Ying Yang, Kai Guo, Zhenlu Sun

**Affiliations:** ^1^Yantai Center for Disease Control and Prevention, Yantai, Shandong, China; ^2^Department of Liver Disease Hepatic, Yantai Infectious Diseases Hospital, Yantai, Shandong, China; ^3^Laiyang Center for Disease Control and Prevention, Yantai, Shandong, China; ^4^School of Information and Electrical Engineering, Ludong University, Yantai, Shandong, China

**Keywords:** infectious diarrhea, pathogen spectrum, surveillance, epidemiological characteristics, Yantai City

## Abstract

**Background:**

Analysis of the pathogenic spectrum, epidemiological characteristics and molecular epidemiological features of important pathogens of infectious diarrhea in Yantai City, Shandong Province, 2018–2019, were analyzed to provide a reference basis for the prevention and control of infectious diarrhea.

**Methods:**

A total of 1514 stool specimens were collected within 3 days of the onset of diarrhea in secondary or higher hospitals in Yantai from 2018–2019, and all specimens were tested for the presence of seven major viruses and three major bacteria by employing reverse transcription ploymeraer chain reaction (RT-PCR). Population and seasonal analyses were also performed on positive samples for infectious diarrhea. The top two ranked rotavirus and norovirus were focused on genotyping and analysis of geographical distribution.

**Results:**

The study showed that the high prevalence of infectious diarrhea in Yantai, Shandong Province, for two consecutive years in 2018 and 2019 was in young children aged 1–5 years, accounting for 48.6% of the total number of cases. Viral diarrhea was distributed throughout the year with no obvious seasonal distribution, while bacterial diarrhea was predominant in summer. Of 1514 stool specimens, the total positive rate of specimens was 43.92% (665/1514). One pathogen was detected in 507 specimens, two pathogens in 107 specimens, and three pathogens in 44 specimens, with mixed infections accounting for 22.71% of positive specimens (151/665). Viral diarrheal pathogens accounted for 93.23% (620/665) of positive samples. The percentages of positive samples for Rotavirus (RV), Norovirus GI, Norovirus GII, Enterovirus universal (EV), Enteroadenovirus (EAdV), Sapovirus (SaV), Astrovirus (Astv), *Salmonella* (SE), *Listeria monocytogenes* (LiMo), and *Vibrio parahaemolyticus* (VP) were 48.57%, 3.61%, 15.34%, and 10.68% of the total positive samples.

**Conclusions:**

This study analyzed in detail the composition of infectious diarrhea pathogen spectrum, pathogen alternation pattern, seasonal distribution and population distribution of pathogens in Yantai City, Shandong Province, 2018–2019, to provide a basis for improving relevant local preventive measures and reducing the disease burden.

## 1. Introduction

Diarrhea is one of the common diseases worldwide. More than 3 bowel movements per day, with thin and soft stools and more than 200 g of stool in 24 h, or <200 g of stool per day but more than 3 times, with symptoms of perianal discomfort, urgency or fecal incontinence are called diarrhea (1). There are two main types of diarrhea: non-infectious diarrhea and infectious diarrhea. Infectious diarrhea, as an important cause of diarrhea, has a high incidence and wide prevalence, and is an important disease that endangers human health (2). As many as 1.5 billion people in developing countries suffer from the disease each year, and a very high proportion of children (3).

Infectious diarrhea is mostly caused by infections including viral, bacterial and parasitic pathogens, among which viruses are the main pathogens causing diarrhea in recent years (4, 5). Diarrhea viruses, especially Norovirus (NoV) and Rotavirus (RV), are the leading causes of gastroenteritis worldwide (6). Adenovirus (AdV), Astrovirus (AstV) and Sapovirus (SaV) are also common viral pathogens.

In China, there are significant differences between developing and developed regions in terms of economic and social characteristics and health and in the incidence of infectious diarrhea. There are significant differences in the incidence of infectious diarrhea in regions with different economic conditions. Over the past two decades, in an effort to reduce the disease burden of diarrhea, the Chinese government has made great strides in improving public livelihoods, and childhood mortality has decreased significantly. Despite many efforts, China is still one of the fifteen countries with a high prevalence of diarrheal diseases worldwide, and to date, diarrheal diseases are among the top 3 notified infectious diseases (7).

With the exception of rotavirus, there are no vaccines available for the prevention of other diarrheal pathogens. Because the clinical manifestations of diarrhea caused by different pathogens are very similar, the pathogen distribution characteristics are not clear, resulting in the pathogens of infectious diarrheal diseases not being accurately diagnosed. The monitoring of infectious diarrhea pathogen distribution and genetic variation is important for the prediction and early warning of infectious diarrhea epidemic trends, clinical diagnosis and treatment, vaccine development and application, and new drug screening. Long-term standardized surveillance of the pathogenic spectrum composition and epidemiological characteristics of infectious diarrhea can provide a basis for the development of prevention and control strategies and the adoption of targeted prevention and control measures.

In recent years, the lack of systematic analysis of the pathogenic spectrum, genotypes and genetic variation of infectious diarrhea (containing both viral and bacterial pathogens) covering the whole area of Yantai City has made it difficult to achieve precise prevention and control, so this study describes the local situation in Yantai using the local situation of a total of 1,514 human diarrhea samples from 2018 to 2019, by testing for ten important infectious diarrhea pathogens (seven major viruses and three major bacteria) for long-term standardized surveillance. Testing Rotavirus (RV), Norovirus G I, Norovirus G II, Enterovirus (EV), Enteroadenovirus (EAdV), Sapovirus (SaV), Astroviru (sAstv), Salmonella (SE) Listeria monocytogene (LiMo), Vibrio parahaemolyticus (VP)clarifed the composition of pathogen spectrum, pathogen alternation pattern, seasonal distribution and population distribution of pathogens, for provide a basis for improving relevant preventive measures and reducing the disease burden.

## 2. Materials and methods

### 2.1. Sample source and sample collection

Both clinical diagnosis and laboratory diagnosis of infectious diarrhea are based on the Diagnostic Criteria for Infectious Diarrhea (WS271–2007) (8). Medical institutions of all levels and types in 13 districts and counties under the jurisdiction of Yantai City, Shandong Province, when suspected cases of infectious diarrhea are detected, they are reported directly on the network in accordance with the “Infectious Disease Information Reporting Management Standards”, this study included an all-age cohort. Those who do not currently have the conditions for direct network reporting should report to the local county-level CDC within 24 h, while the completed infectious disease report card should be sent within 24 h. Each county-level disease control agencies monthly from the receipt of case reports, in order to the first five cases should be reported within 48 h after the case investigation (<5 cases, all investigations), complete the “case investigation form for surveillance of viral diarrhea cases”.

Medical institutions at all levels in 13 districts and counties under the jurisdiction of Yantai City conducted case investigation of the first 5 cases while collecting stool specimens according to the arrangement of local CDC institutions. Stool specimens were collected from a total of 1,514 cases of clinically infected diarrhea patients in Yantai for 2 consecutive years from 2018 to 2019. 3 to 5 g of the specimen were collected from each case and placed in sterile, dry collection tube, preferably without the use of a glass container. No protective agents, culture media, etc. should be added to the container in advance. Stool specimens collected should not be diluted in advance, and anal swabs should not be collected as much as possible. Antibiotics were given after the collection of samples. Samples were refrigerated at 4°C after collection, sent to the laboratory within 24 h, stored frozen at −80°C, and tested centrally to avoid repeated freezing and thawing.

### 2.2. Extraction of total fecal nucleic acid

Total nucleic acid was extracted from 1,514 clinical specimens. Mung bean size feces or 50 ~ 100μl water feces were extracted and 500μl normal saline was added to make 10 ~ 20% suspension. After centrifugation at 9100 g for 5 min, 200 μl supernatant was obtained. Shengxiang automatic nucleic acid extraction instrument and kit were used for nucleic acid extraction of the treated specimens, and the operation was carried out in accordance with the kit instructions.

### 2.3. Detection of pathogenic bacteria in diarrhea

The extracted nucleic acids were used for the detection of ten diarrheal pathogens using the preformed plates produced by Shanghai Berger Biotechnology Co. (VP) by real-time fluorescence PCR. Specific methods:After sample nucleic acid extraction, the membranes of the preformed PCR plates were lifted, and 5 μl each of the treated specimen nucleic acid to be examined, negative control and positive control were added, respectively, and the membranes were sealed. Amplify by instantaneous low-speed centrifugation followed by placement of a fluorescent PCR detector. The amplification curve was S-shaped, and CT.

## 3. Results

### 3.1. Infectious diarrhea pathogen surveillance in Yantai City, Shandong Province, 2018 and 2019

#### 3.1.1. Crowd distribution

From January 1 to December 31, 2018, a total of 779 cases of diarrhea were registered in the diarrhea surveillance system in Yantai, Shandong Province. Subjects were aged 0–89 years, of which 438 cases were male and 341 cases were female, with a male-to-female ratio of 1.28:1.

From January 1 to December 31, 2019, the total number of diarrhea cases registered in the diarrhea surveillance system in Yantai, Shandong Province, was 735. Subjects were aged 0–89 years, including 405 males and 325 females, with a male-to-female ratio of 1.24:1.

#### 3.1.2. Pathogenic spectrum of infectious diarrhea in Yantai, 2018 and 2019

**Figure 1:**
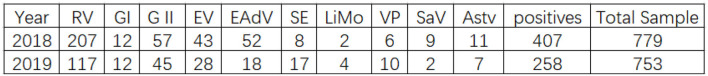
Pathogenic spectrum of infectious diarrhea in Yantai, China in 2018 and 2019.

As shown in Figure 1, the total positive rate of specimens was 52.24% (407/779) out of 779 stool specimens. The number of samples detected infected with 1 pathogen was 301, those infected with 2 pathogens was 72, and those infected with 3 pathogens was 34, with mixed infections accounting for 26.04% of the positive specimens (106/407). Viral diarrheal pathogens accounted for 96.07% of the positive samples. The percentages of positive samples for rotavirus, norovirus GI, norovirus GII, enterovirus general (EV), enteric adenovirus (EAdV), zapovirus (SaV), astrovirus (Astv), salmonella (SE), listeria monocytogenes (LiMo), and vibrio parahaemolyticus (VP) to the total positive samples were 50.86, 2.95, 14.00, and 10.57%, respectively.

As shown in Figure 1, the total positive rate of specimens was 35.10% (258/735) out of 735 stool specimens. The number of specimens infected with one pathogen was 206, the number of samples infected with two pathogens was 35, and the number of samples infected with three pathogens was 10, with mixed infections accounting for 17.44% of positive specimens (45/258). The viral diarrheal pathogens accounted for 89.46% of the positive samples. The percentages of positive samples for rotavirus (RV), norovirus GI, norovirus GII, enterovirus general (EV), enteric adenovirus (EAdV), sapovirus (SaV), astrovirus (Astv), salmonella (SE), listeria monocytogenes (LiMo), and vibrio parahaemolyticus (VP) were 45.00, 4.62, 17.31, and 10.77% of the total positive samples, respectively.

#### 3.1.3. Seasonal distribution of the ten pathogens causing infectious diarrhea in Yantai in 2018 and 2019

The seasonal distribution of infectious diarrhea pathogens in 2018 and 2019 had a similar pattern to that of 2017 (9). Viral infections were distributed throughout the year, with peaks of rotavirus (RV) and astrovirus (Astv) infections mainly occurring in winter and spring, and no obvious seasonal pattern for enterovirus general (EV) and norovirus (G I and G II) infections. Bacterial infections were more seasonal than viral infections, showing a peak of infection in summer, with peaks of pathogens such as Salmonella (SE), Listeria monocytogenes (LiMo), and Vibrio parahaemolyticus (VP) occurring in July-September.

**Figure 2:**
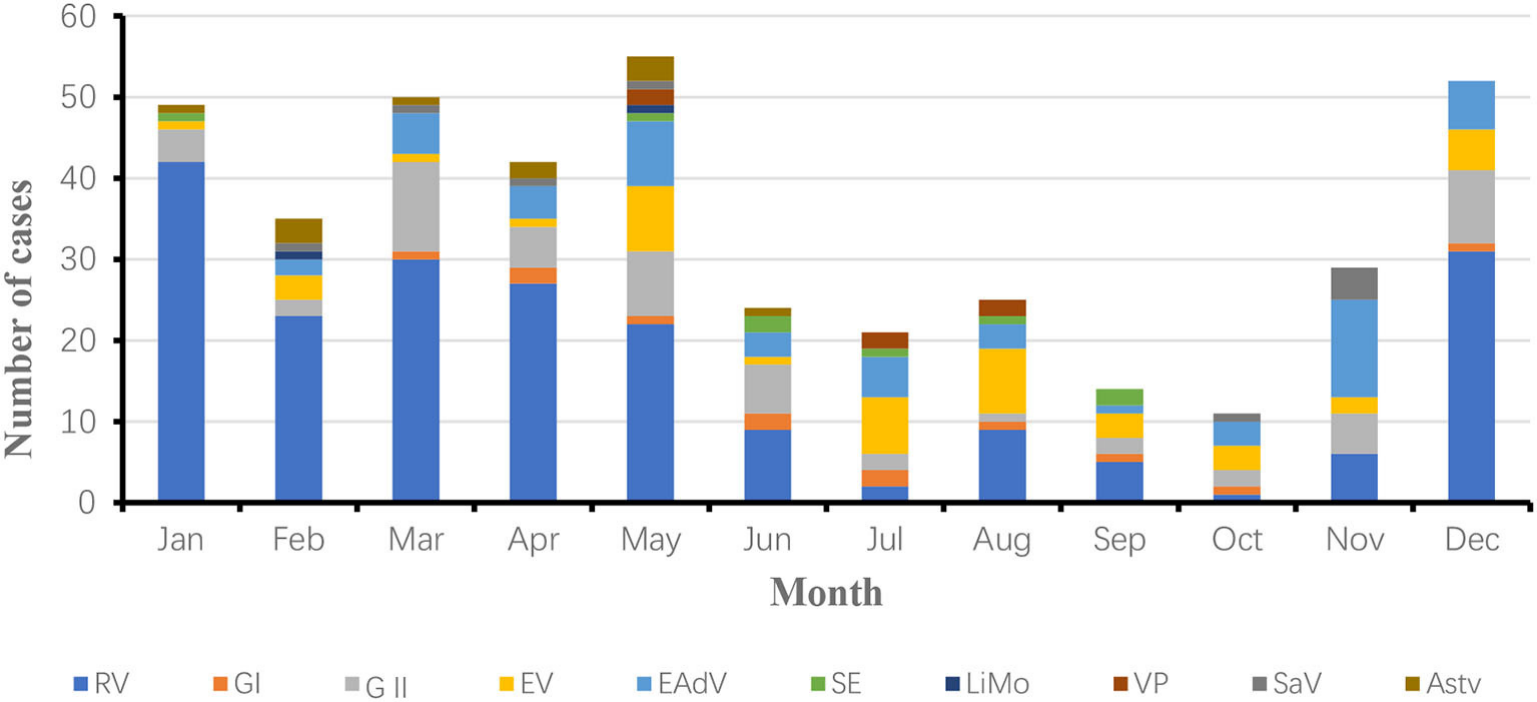
Seasonal distribution of ten pathogens of infectious diarrhea in Yantai, China, 2018.

**Figure 3:**
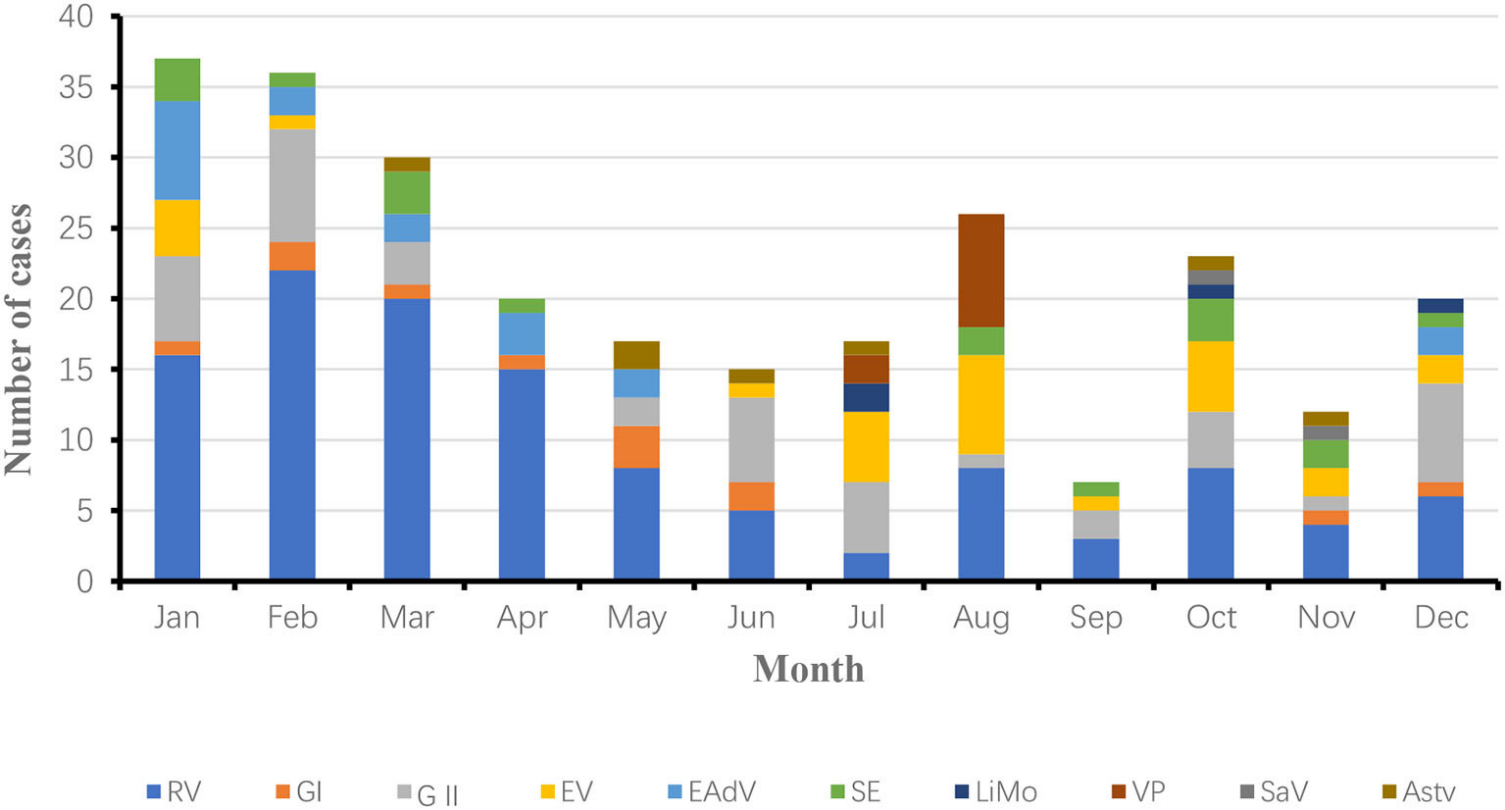
Seasonal distribution of ten pathogens of infectious diarrhea in Yantai, China, 2019.

As seen in the Figure 2, the number of cases was relatively high in January, March, April, May and December and relatively low in September and October in 2018. The main reason for this is the higher distribution of the dominant pathogen rotavirus in winter and spring. As seen in the Figure 3, the number of cases was relatively high in January, February, March, and August and relatively low in September and November in 2019. The high number of cases in January, February and March was mainly due to the high incidence of rotavirus and norovirus, and the high number of cases in August was mainly due to the high number of rotavirus, Vibrio parahaemolyticus and enterovirus generalist.

#### 3.1.4. Age distribution of diarrhea patients infected with ten pathogens in Yantai City, 2018 and 2019

The age of the patients with diarrhea in this study was 0–89 years. They were divided into 6 groups, namely, infants and children aged 0–1 year, infants and children aged 2–5 years, adolescents aged 6–18 years, adolescents aged 19–30 years, middle-aged people aged 31–60 years and older adult people aged 61–89 years, the age of onset of infectious diarrhea was mainly concentrated in infants and children under 1 year of age in 2018 and 2019.

**Figure 4:**
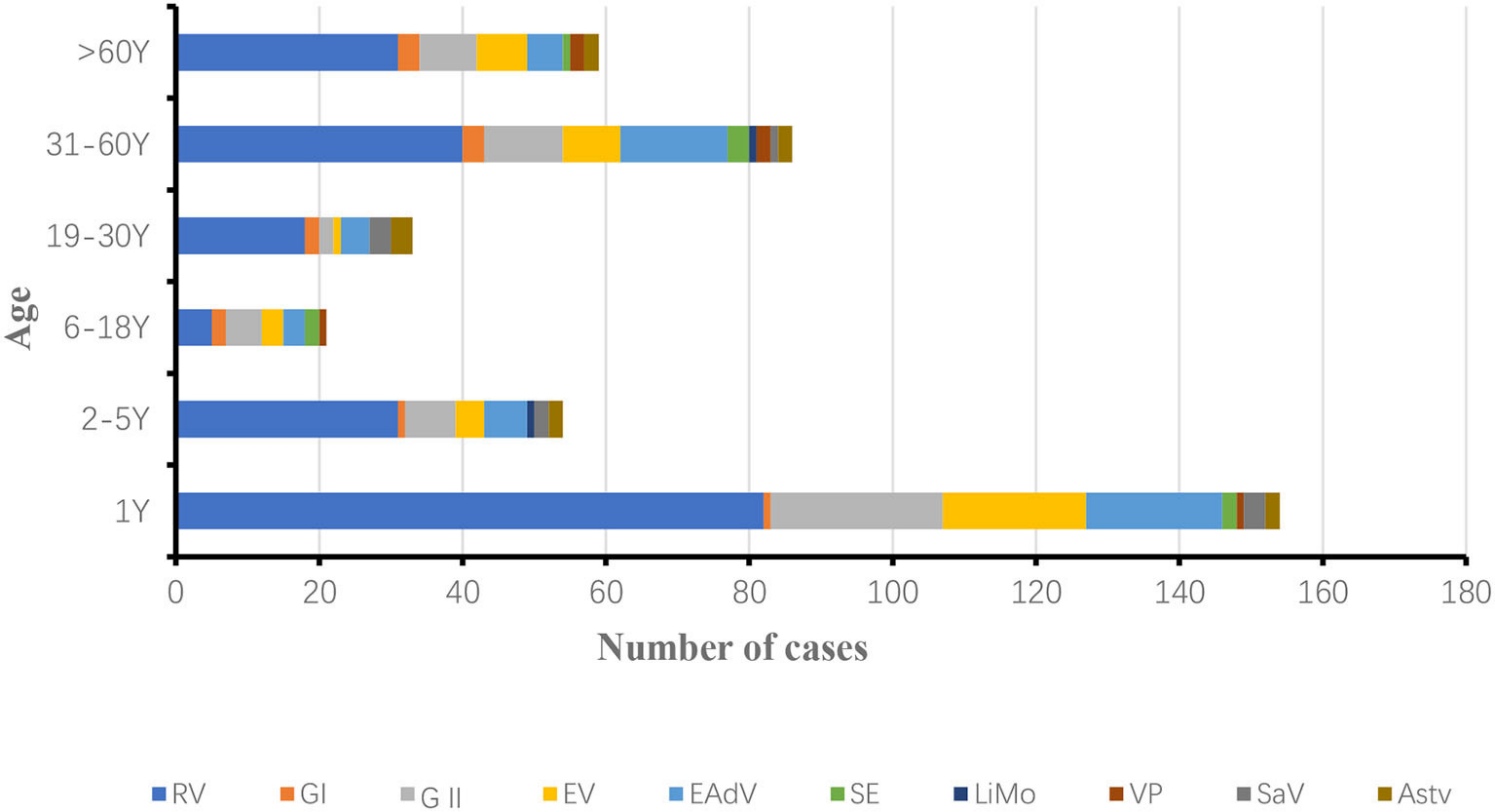
Age distribution of diarrhea patients infected with ten pathogens in Yantai, China, 2018.

In 2018, 154 positive samples were detected in patients under 1 year of age, accounting for 37.83% (154/407) of the total number of patients with diarrhea. RV, NoV, EV, LiMo and EAdV positivity rates were significantly higher in patients under 1 year of age than in other age groups. The incidence of infectious diarrhea was lowest in the 6–18 years age group, rotavirus infection was lowest in the 6–18 years age group, and the most complex combination of pathogens was found in the 19–30 years age group (Figure 4).

**Figure 5:**
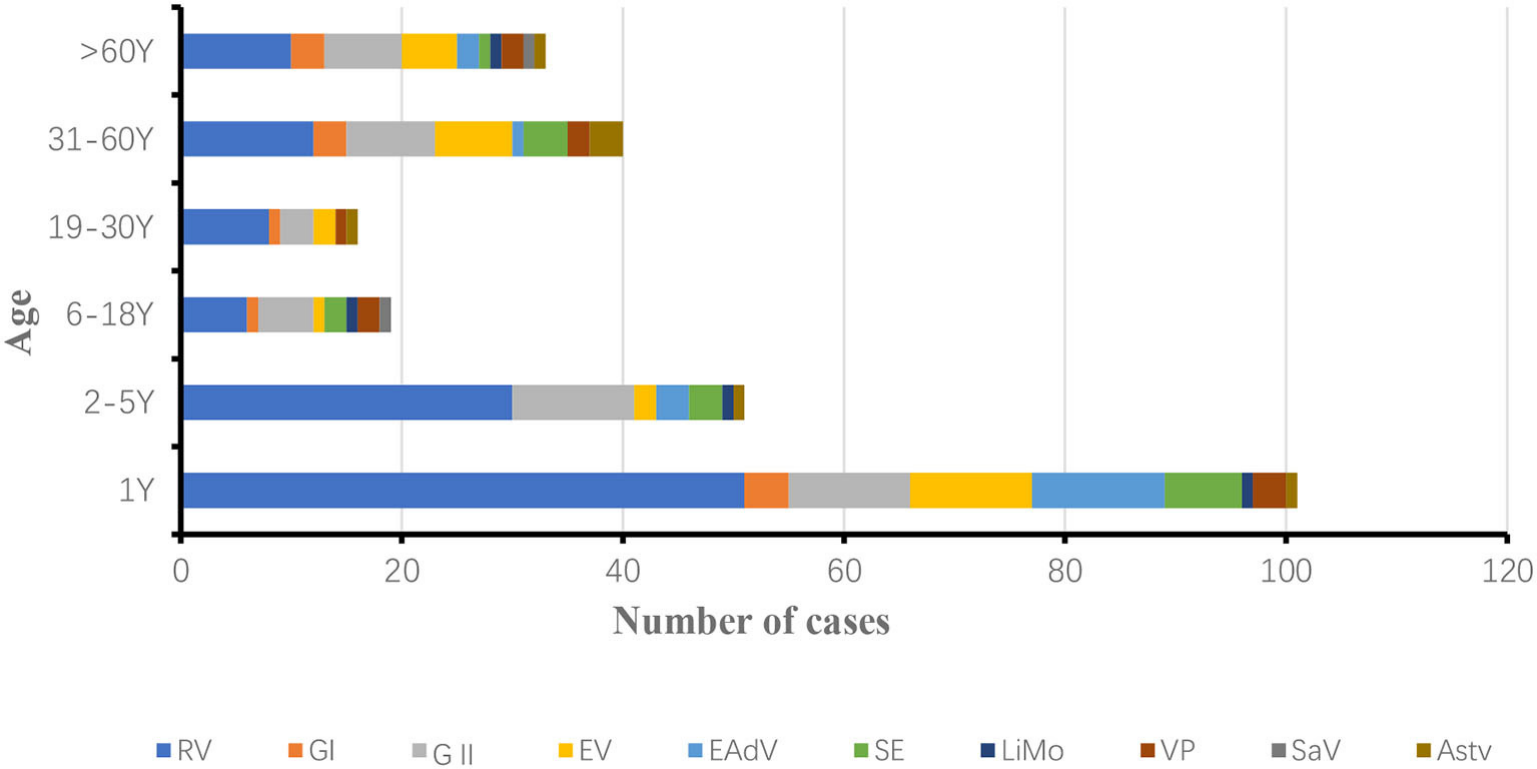
Seasonal distribution of ten pathogens of infectious diarrhea in Yantai, China, 2019.

In 2019, 100 positive samples were detected in patients under 1 year old, accounting for 38.76% (100/258) of the total number of patients with diarrhea. rotavirus RV, NoV, EV, LiMo, SE and EAdV positivity rates were significantly higher in patients under 1 year old than in other age groups. The incidence of infectious diarrhea was lowest in the age group 6–30 years, and the incidence of rotavirus was lowest in the age group 6–18 years, with similar pathogen categories in all age groups (Figure 5).

### 3.2. Distribution of rotavirus and norovirus in various districts of Yantai

Rotavirus (RV) accounted for the highest percentage of positive samples in 2018 at 50.86%, followed by norovirus (NoV) at 16.95%. rotavirus (RV) accounted for the highest percentage of positive samples in 2019 at 45.00%, followed by norovirus (NoV) at 22.93%.

In order to better understand the epidemiological patterns of the two pathogens for precise guidance of prevention and control, a study was conducted on the distribution of these two pathogens in each district of Yantai city. It was found that the distribution patterns of rotavirus (RV) and norovirus (NoV) in Yantai districts were similar for three consecutive years, with the total infections in Zhifu, Laiyang, Penglai, and Haiyang being significantly higher than those in other districts, and Longkou having the lowest infection rate each year.

## 4. Discussion

Pathogenic surveillance helps to track the composition and changes in the pathogenic spectrum and has important public health significance for the prevention and control of infectious diarrhea. The purpose of this study was to conduct systematic surveillance of human infectious diarrheal diseases in Yantai, Shandong Province, 2018–2019, to clarify their epidemiological characteristics, etiology and pathogen composition, and to explore and develop targeted prevention and control policies for infectious diarrheal diseases in the region.

This study was based on the detection of 1,514 human diarrhea samples for two consecutive years from 2018 to 2019 in Yantai City for ten important pathogens of infectious diarrhea, analyzing their pathogenic spectrum composition, pathogen alternation patterns and population distribution characteristics, elucidating the main pathogens of infectious diarrhea epidemic in Yantai City and their variation and change patterns, epidemiological characteristics and risk factors, and providing a basis for the prediction and early warning of the epidemic.

The results of the study showed that the total positivity rate of ten pathogens in Yantai City for two consecutive years 2018–2019 was 52.54 and 35.10%, respectively, with viral diarrhea pathogens accounting for 96.07 and 88.76% of the total positive specimens, respectively, similar to the results reported in other regions of China where viral diarrhea predominates, and slightly higher than the 60–75% reported in other regions (10, 11). It was found that the composition structure of the pathogen spectrum in Yantai was basically the same for three consecutive years, with the top five dominant pathogens in order: rotavirus, norovirus, enterovirus generalist, enteric adenovirus, and astrovirus. The top two pathogens in terms of infection rate, rotavirus (RV) and norovirus (NoV), accounted for more than 60% of infectious diarrhea, which is basically consistent with the surveillance results in other regions of China (12, 13).

In recent years, with the continuous updating of research tools and studies, the problem of mixed infections, which contain mixed infections between viruses, mixed infections between bacteria, and cross-infection between viruses and bacteria, has received increasing attention. The results of the study showed that mixed infections of pathogens in diarrhea samples in Yantai accounted for 26.04 and 17.44% of positive specimens in 2018–2019, respectively. It has been pointed out that mixed infections of different pathogens may have synergistic effects, which may lead to worsening of diarrhea, the blood group antigen (HBGA) produced by bacteria can form complexes with viruses (14), enhance its stability and ability to enter cells susceptible to viral replication (15, 16). It is also possible that the reduced immunity of the organism in patients with diarrhea facilitates infection by other pathogenic bacteria. So more attention should be paid to mixed infections in future studies. From the results of two consecutive years of surveillance in Yantai, it was found that the distribution of various pathogens had certain seasonal patterns, and bacterial infections showed obvious seasonality, with infections of bacterial pathogens such as Salmonella (SE), Listeria monocytogenes (LiMo), and Vibrio parahaemolyticus (VP) peaking in summer and autumn, consistent with the results of related studies (17, 18). On the other hand, viral infections are distributed throughout the year, with no specific temporal distribution characteristics for norovirus (NoV) and enterovirus generalist (EV), high prevalence of rotavirus (RV) throughout the year, especially peaking in winter and spring, and enteric adenovirus (EAdV) infections more frequently in summer, suggesting attention to high prevalence pathogenic infections in different seasons (19–21). Meanwhile, the results of the study showed that the number of cases of infectious diarrhea caused by ten pathogens in 2018 and 2019 varied considerably in different seasons, and synthesizing the seasonal distribution of pathogens in both years, it can be seen that rotavirus and norovirus are the dominant pathogens of infectious diarrhea in Yantai, and the overall number of cases is high in months with high distribution of these two pathogens. The incidence of Vibrio parahaemolyticus in August 2019 The number was significantly higher than in previous years, resulting in a higher total number of cases in this month of the year. Vibrio parahaemolyticus, as a highly prevalent pathogen in coastal areas, is mainly found in seafood and should therefore be given priority for prevention and control in summer.

The surveillance population in this study was an all-age group population ranging from 0 to 89 years old for two consecutive years, and was divided into six groups according to total age, which helped to understand the etiologic composition of infectious diarrhea in all age groups in Yantai, Shandong Province. The results of two consecutive years of surveillance showed that the prevalence of male patients was slightly higher than that of females, and it was also found that positive patients in infectious diarrhea were mainly concentrated between 0 and 1 year of age, which is consistent with the results of domestic and international studies (22–24). The occurrence of this condition may be related to the underdevelopment of the infant's immune system and feeding practices, as well as the large proportion of rotavirus, which is an important pathogen causing severe diarrhea in infants and children. Because rotaviruses are highly variable and pathogenic and there are no specific drugs available, the development and promotion of rotavirus vaccines is currently the primary measure to prevent and control rotavirus infections (25, 26). The results of two consecutive years of testing found the lowest incidence of rotavirus in the 6–18 years age group, probably because this age group is the primary and secondary school stage, with improved immunity and good personal and dietary hygiene at school and at home. In addition, in China, older children and adults usually do not actively seek medical attention when they have diarrhea, preferring to purchase their own medication. Since China's statutory infectious disease reporting information system is a passive surveillance network, only those who actively seek medical care are included in the surveillance system. This is one of the reasons for the high proportion of reported cases in young children.

Although diarrheal symptoms caused by norovirus are usually self-limiting, there has been a significant increase in domestic aggregations and outbreaks in recent years, and therefore a key concern (27). It was found that there were age differences in the prevalence of Vibrio parahaemolyticus, with the highest rate of Vibrio parahaemolyticus positivity in the age group of 19–60 years. This phenomenon may be related to the serious contamination of Vibrio parahaemolyticus in seafood such as shellfish, fish, and shrimp in coastal areas of Yantai, where young adults tend to consume raw or semi-raw aquatic products (28), while younger and older people are less likely to use them. Therefore, strengthening food safety supervision and promotion of healthy eating habits will play a positive role in reducing the incidence of Vibrio parahaemolyticus.

The monitoring results show that rotavirus and norovirus are the main causes of infectious diarrhea in Yantai, accounting for more than 50%. Therefore, a focus was conducted to analyze the distribution of these two pathogens in the thirteen-county urban area under the jurisdiction of Yantai City, Shandong Province, for three consecutive years. The results showed that there were significant differences between different areas of Yantai city. An analysis of the possible causes of this result showed that the total infections in Zhifu District, Laiyang City, Penglai City and Haiyang City were significantly higher than those in other areas, and Longkou City had the lowest infection rate each year. Zhifu District, as the core district of Yantai City, is characterized by a dense population and a high degree of population mobility. Penglai district and Haiyang city are coastal areas with convenient transportation and more raw seafood eating. Laiyang city belongs to the less economically developed area of Yantai, so environmental hygiene and eating habits may be problematic. In summary, the high incidence of these two pathogens may be related to factors such as dense regional population, convenient transportation, high population mobility, dietary habits, and economic situation. The low infection rate in Longkou, a more developed area of Yantai, may be due to better environmental hygiene, dietary habits, and high quality of the population. The results of this study guide us more precisely to take different preventive and control measures for different areas to reduce the incidence of infectious diarrhea more effectively.

Rotavirus vaccine is the most economical and effective means to prevent rotavirus infectious diarrhea. Currently, there are two types of rotavirus vaccines available in China: live oral rotavirus vaccine and live oral pentavalent reassorted rotavirus attenuated vaccine, of which the live oral rotavirus vaccine is mainly used to prevent diarrhea caused by rotavirus in infants and children of group A (G1), and the live oral pentavalent reassorted rotavirus attenuated vaccine is mainly used to prevent diarrhea in infants and children caused by serotype A group (G1, G2, G3, G4, G9). Therefore, it is recommended that children under 3 years of age should be immunized once a year to achieve better immunization effect. Our results showed that the genotyping results of RV in Yantai from 2017 to 2019 were all dominated by G genotype G9, all P genotypes were dominated by P (8), and the G/P combination was dominated by even G9P (8). 60.84% of the total RV samples tested were G9P (8) in 2017, 94.69% in 2018, and 83.76% (29). The results of this study reveal the epidemiological characteristics of rotavirus infection and the development pattern of the dominant type in Yantai in recent years, and also provide guidance for the selection of local rotavirus vaccine, suggesting that infants and children in Yantai should preferentially receive vaccines containing the G9 type in recent years.

With the exception of rotavirus, there is a lack of relevant preventive vaccines for other pathogens, so the control of diarrheal cases relies heavily on the use of antibiotics. However, the misuse of antibiotics has become a serious problem in China, so the study of the composition of the pathogenic spectrum of diarrheal diseases and the pattern of pathogen alternation is a positive guide for the diagnosis and treatment of the disease, and is a very valuable public health intervention. The results of the study suggest that Yantai City, Shan Province, should focus on the prevention and treatment of bacterial diarrhea in summer and viral diarrhea, especially rotavirus and norovirus infections, throughout the year. Infectious diarrhea in Yantai has the highest rate of infection in children under 1 year of age, and studies have shown that 63% of global diarrhea cases occur in children under 5 years of age (24), and because the etiology of diarrhea in children is constantly evolving, it is particularly important to analyze its pathogenic spectrum, and studies have shown that rotavirus occupies a large proportion, and because vaccines are the most effective means of reducing rotavirus infection rates, relevant authorities should actively advocate rotavirus vaccination for infants and children under 5 years of age, and the management of childcare institutions and schools should be strengthened to pay attention to food hygiene and dietary habits (30) to reduce the incidence and economic burden of diarrhea.

In summary, surveillance of infectious diarrhea should be continuously strengthened to provide accurate epidemiological information for clinical diarrheal diseases and to reduce the incidence, economic burden, and impact on patients, families, and society. The relevant authorities should pay attention to strengthening health education for the whole population, enhancing food hygiene supervision, reforming and improving environmental hygiene, and advocating healthy dietary habits and healthy lifestyles for the whole population.

There are some limitations in this study. In future studies we will perform detailed genotyping of norovirus, sarvovirus, and astrovirus to further refine the pathogenic profile, analyze genomic features, and possibly identify new or rare pathogens that cause infectious diarrhea.

## Data availability statement

The original contributions presented in the study are included in the article/supplementary material, further inquiries can be directed to the corresponding authors.

## Ethics statement

The studies involving human participants were reviewed and approved by the National Institute for Viral Disease Control and Prevention. Written informed consent to participate in this study was provided by the participants' legal guardian/next of kin.

## Author contributions

QG: data curation, methodology, investigation, and writing of the original draft. HL: data curation, formal analysis, investigation, and resources. WY: formal analysis, investigation, and methodology. ZW: data curation. YY, KG, and ZS: methodology, data curation, writing—review and editing, and project administration. All authors contributed to the article and approved the submitted version.
